# Association of cardiovascular health with cognitive function and the mediating effect of depressive state

**DOI:** 10.3389/fnagi.2024.1465310

**Published:** 2024-12-18

**Authors:** Yiwei Hu, Xuelu Sun, Chen Guo, Ruiyun Wu, Jiahao Dou, Shoufang Song, Fanshun Guo, Jin Wei

**Affiliations:** ^1^Department of Cardiology, The Second Affiliated Hospital of Xi’an Jiaotong University, Xi’an, Shaanxi, China; ^2^Clinical Research Center for Endemic Disease of Shaanxi Province, The Second Affiliated Hospital of Xi’an Jiaotong University, Xi’an, Shaanxi, China

**Keywords:** cardiovascular health, cognition function, Life’s Essential 8, depression state, NHANES

## Abstract

**Background:**

Although previous research has substantiated lifestyle and cardiovascular-related measures have some impact on cognitive function, studies focusing on the correlation between Life’s Essential 8 (LE8), an indicator for quantifying cardiovascular health (CVH), and cognitive function are limited. Consequently, this study sought to explore the potential link between CVH and cognitive function as well as to determine if depressive states mediated the relationship.

**Methods:**

A total of 2,263 individuals were selected from the 2011–2014 National Health and Nutrition Examination Survey (NHANES). Post-averaged LE8 scores was classified as low CVH (0–49), moderate CVH (50–79), and high CVH (80–100) according to the American Heart Association (AHA). Cognitive function was evaluated using the Animal Fluency Test (AFT), the Digit Symbol Substitution Test (DSST), the instant recall test (IRT), and the delayed recall test (DRT). The Z-score is figured by subtracting the average of the scores of four test sections and dividing by the standard deviation. Models of multi-variable linear regression were employed to appraise the relationships between CVH and the Z-score for cognitive function. Depression was assessed through the utilization of the Patient Health Questionnaire (PHQ-9). Points of 10 or above indicated a positive diagnosis. Weighted linear regression and restricted cubic spline (RCS) were employed to evaluate the correlation between CVH and cognitive function. Pearson’s test was utilized to explore the interrelation among primary variables and mediated effects analyses of depressive states.

**Results:**

A significant positive linear relationship was observed between LE8 score and cognitive function Z-score. In all models, there was a positive correlation between higher Z-score for cognitive function and every ten points added to the LE8 score, which evaluates CVH. The findings of the mediating effect study indicated that the effects of cardiovascular health on cognitive function were partially mediated by depression.

**Conclusion:**

Results showed a meaningful positive linear correlation between the level of CVH and cognitive function, with a mediating role for depression. These results accentuate the significance of sustaining high CVH and avoiding depression to improve cognitive functioning.

## 1 Introduction

In the World Health Statistics Report 2023, the number of deaths and disease burden caused by cardiovascular diseases (CVDs) remain in the first place (World Health Organization, 2023). Cardiovascular health (CVH) is of paramount importance in reducing mortality and morbidity rates worldwide, with poor CVH leading to severe outcomes like myocardial infarction and cerebrovascular accident. Furthermore, the health of the cardiovascular system has extensive implications beyond physical health, influencing mental health, cognitive function, and overall quality of life (Wei et al., 2023; Xian et al., 2024). In order to gain a comprehensive understanding of cardiovascular health, the American Heart Association (AHA) proposed a revised definition “Life’s Essential 8 (LE8),” including two primary categories: health behaviors and health factors. The health behaviors domain contains diet, physical activity, nicotine exposure, and sleep health, while the health factors domain includes body mass index (BMI), non-high-density lipoprotein (non-HDL) cholesterol, blood glucose levels, and blood pressure (Donald et al., 2022). According to the release of the President’s recommendations, LE8 score range from 0 to 100, with higher scores indicating better CVH. The value and reliability of the indicator have been fully confirmed by the numerous studies that show a strong negative correlation between the endpoints of non-cardiovascular disease, all-cause mortality, and the incidence of various CVDs as measured by this indicator (Bundy et al., 2021). The updated LE8 also takes into account individual and age-related variations, rendering it more applicable to public health initiatives (Lloyd-Jones et al., 2022).

Previously, it was believed that cardiovascular disease posed little risk to the brain (Lassen, 1964). Conversely, there is increasing evidence indicates that cardiovascular health is inextricably linked to brain function (de Leeuw et al., 2004, den Heijer et al., 2005). Cognitive function is a kind of advanced function of brain to process information, assess and interpret external stimuli, and execute complex mental tasks. As population aging intensifies worldwide, cognitive decline associated with aging has gradually become a primary public health problem (Lassi et al., 2023). Cognitive impairment is significantly associated with diseases such as dementia and depression, which has unfavorable effects on the health and quality of life of elderly people (Denver and McClean, 2018). Moreover, studies have shown that several cardiovascular diseases are risk factors for cognitive decline, and the two interact and influence each other (Paul et al., 2005, Schievink et al., 2022). Therefore, it is necessary to further study the mechanism between cognitive decline in old age and CVH in order to propose effective preventive measures.

Depression is acknowledged as one of the primary causes of disability globally, making a significant contribution to the total global illness burden (Malhi and Mann, 2018). Several longitudinal studies and meta-analyses have shown an association between depression and CVD (Frasure-Smith et al., 1993; Lespérance et al., 2002; Vicario and Cerezo, 2023). Of note, clinical studies have found that 85–94% of patients with depression have cognitive dysfunction at the time of onset, and 39–44% still have cognitive dysfunction after remission of depression (Conradi et al., 2011), suggesting that there is some relation between depression and cognitive function (Lim et al., 2013). Besides, there are few studies on the relationship among CVH and cognitive function and depression state in the population. This study received some support from earlier research on the mediating role of depression in the association between an active lifestyle and cognitive function in the elderly (Xian et al., 2024).

In view of the above, the purpose of this study was to examine the relationship between CVH levels and cognitive function, as well as the impact of depression on cognitive abilities. The goal is to provide more precise and valuable information for measures that can enhance cognitive function, thereby improving the health and quality of life for the elderly population.

## 2 Materials and methods

### 2.1 Study design and participants

The National Health and Nutrition Examination Survey (NHANES) is a comprehensive program of studies. This cross-sectional study employed data from the 2011–2014 NHANES cycles and participants completed informed consent forms.

The 2011–2014 survey yielded results from 19,931 participants. Initially, we removed individuals with incomplete data in the eight scoring items that defined the LE8 score. We also excluded those who did not complete the cognitive function assessment. The study included participants who displayed complete scores for both measures. Among them, 8,082 participants had the eight scoring indicators of LE8, and 2,934 participants had the cognitive function Z-score data. Finally, 2,263 participants with LE8 scores and Z-score for cognitive function and other required indicators were included in the statistical analysis.

### 2.2 Demographic characteristics

The basic characteristics of population were collected by questionnaires during the home interview. In this study, age was divided into three groups: 60–69 years, 70–79 years, or ≥ 80 years. Race/ethnicity was categorized as Mexican American, Hispanic, non- Hispanic (NH) White, NH Black, and Other. The poverty ratio was calculated as the ratio of monthly household income to poverty levels and sorted into 3 groups: < 1.3 (low income), 1.3–3.5 (middle income), and > 3.5 (high income). Less than ninth grade, ninth to eleventh grade, high school graduate/GED or equivalent, some college/AA degree, college graduate/above, and don’t know where the categories used to classify education levels. Marital status was classified as coupled and single.

### 2.3 Measurement of LE8

As mentioned earlier, LE8 updated model encompasses eight key components and each integral to maintaining and enhancing heart function. According to the AHA, each indicator is given a new scoring algorithm with a score ranging from 0 to 100. The total score is then divided by 8 to derive a comprehensive cardiovascular health score, also ranging from 0 to 100. Supplementary Table 1 provides a detailed explanation of how the scores for each LE8 measure were calculated using NHANES data. In this study, LE8 score was calculated for each participant based on detailed score calculation tables published in the past (Lloyd-Jones et al., 2022). Participants with a LE8 score of 0–49 were deemed low CVH; 50–79, moderate CVH; and 80–100, high CVH.

The 2015 version of the Healthy Eating Index (HEI) assessed diet metrics (Krebs-Smith et al., 2018). HEI-2015 was calculated by combining the dietary intake data for the corresponding cycle in the Food Pattern Equivalents Database (FPED) with the nutrient intake from the dietary data for the corresponding cycle in the NHANES database, using the Dietaryindex package in the R language. Data on medication history, amount and kind of physical activity, sleeping and smoking patterns, and diabetic condition were gathered using self-report questionnaires. The self-reported frequency of exercise activity was multiplied to get the physical activity and the metabolic equivalent (MET) of each exercise as an indicator of the participant’s exercise status. Measurements of height, weight, and blood pressure were checked up during medical examination. And BMI values were derived from information on height and weight. Results for lipids, blood sugar and glycosylated hemoglobin are available from laboratory data for the corresponding year.

### 2.4 Measurements of cognitive function Z-score

The cognitive component of the NHANES 2011–2014 primarily consists of three tests: the Consortium for the Registry of Alzheimer’s Disease (CERAD) Word Learning and Recall Module, the Animal Fluency Test (AFT), and the Digit Symbol Substitution Test (DSST). The CERAD Word Learning Subtest (CERAD W-L) evaluates the capacity to acquire new linguistic information in the Memory domain through immediate and delayed recall (Morris et al., 1989). It involves three consecutive learning trials in a row and one delayed recall. The reliability of these tests has been proven (Shi et al., 2023). The task for participants is to read out ten unrelated words aloud and then try to remember as many of those words as they can. Throughout the learning trials, the word order is altered. In the AFT, participants are given a minute to identify as many animals as they can and each species they name earns them one point, which is used to evaluate categorical verbal fluency (Balzano et al., 2006). The DSST, a component of the Wechsler Adult Intelligence Scale (WAIS III), evaluates working memory, sustained attention and processing speed (Jaeger, 2018). Participators use a paper form to quickly match symbols to numbers in 133 boxes containing nine numbers and symbols. The sum of all the right matches determines the score. To integrate the results from these distinct cognitive tests, we employed Z-score, which standardize the raw scores by accounting for the population mean and standard deviation. The Z-score is calculated as follows: $Z=\frac{\left(X-\mu \right)}{\sigma }$. Where *X* is the participant’s score, μ is the population mean, and σ is the standard deviation. By converting test scores to Z-score, we eliminate differences in test scaling and ensure that each test contributes equally to the overall cognitive assessment (Wei et al., 2023). A higher score represents better cognitive function.

### 2.5 Measurements and definition of depressive state

The evaluation of depression diagnosis and severity was carried out using the PHQ-9, which comprises nine items with scores ranging from 0 to 27 (Kroenke and Spitzer, 2002). A score exceeding 10 indicates clinically significant depression, suggesting symptoms that fall into the category of moderate to severe.

### 2.6 Statistical analyses

Firstly, in consideration of the intricate sampling design of NHANES, this study takes a methodology that incorporates sample weights and stratification in analysis to make the resulting estimates nationally representative. Weighted means and their accompanying standard deviation (SD) were provided for normally distributed continuous variables in the findings, whereas numerical values and corresponding weighted proportions were used to describe categorical variables.

This study investigated the relationship between independent variable CVH (low, moderate and high CVH groups or per 10-point increment) and dependent variable cognitive function. Rate estimates expressed as β with 95% confidence intervals (CIs) were calculated using weighted linear regression models. Model-1 of the weighted linear regression was adjusted for various demographic variables, such as age, gender, race and ethnicity, marital status, poverty ratio, and educational level. Model-2 included these adjustments and also added the depression symptoms. According to existing theories and literature support, confounder pair adjustment of the mentioned variables is helpful to reduce bias and obtain a more accurate and true relationship between CVH and cognitive function.

The survival rate of the subjects was measured over the study period using Kaplan–Meier curves, and they were grouped according to their levels of cardiovascular health to compare the effect of CVH on survival. The change in time and the probability of survival between different CVH groups were demonstrated.

A subgroup analysis was conducted to investigate potential differences in the effects of CVH on cognitive function among different population groups. The analysis included stratification by age level, gender, race and ethnicity, poverty ratio, marital status, and depressive state. The significance of interactions was estimated using the *P*-values for the production terms between CVH (levels or continued points) and the stratification components.

A regression model utilizing weighted ordinary least squares was utilized to examine the correlation between continuous LE8 score and cognitive function. The number and position of nodes in restricted cubic spline (RCS) were selected under the guidance of the Akaike information criterion in order to achieve an optimal balance (Johannesen et al., 2020).

Pearson’s correlation analysis was utilized to assess the bivariate relationships among CVH, depressive state and cognitive function.

Causal mediation analysis was utilized to explore the potential mediating effect of depression on the association between CVH and cognitive function. The mediation analysis was conducted using the “mediation” package in R, which is widely recognized for conducting such analyses in health research. The mediation analysis provides estimates for the average causal mediation effect (ACME), which captures the indirect effect, and the average direct effect (ADE), representing the direct pathway. We used bootstrapping to compute confidence intervals for both ACME and ADE, ensuring the results are statistically robust.

Data analyses were conducted using R and R Studio software, version 4.3.2. Statistical significance was determined with a *p*-value less than 0.05, applying a two-sided test.

## 3 Results

### 3.1 Population baseline characteristics

The study comprised 2,263 participants aged 60 and above, representing 43.67 million US residents. The weighted mean age of the sample was 69.06 years (6.60), with 1,155 individuals (weighted percentage [WP] 53.7%) identified as women. The majority of participants were identified as NH White (81.8%), had some college or an Associate’s degree (28.8%), were from middle-income groups (72.3%), and were in a coupled relationship (58.4%). The weighted means for the LE8 score and cognition function Z-score were 71.32 (13.01) and 1.10 (3.02), respectively. Moreover, 9.0% of participants exhibited depressive symptoms at a moderate or higher level. The weighted percentages of low, moderate, and high CVH were 5.95, 65.0, and 29.05%, respectively (Table 1). Those participants with moderate or low CVH were more likely to be older, male, Mexican American, single and alone, as well as to have a lower education level, when compared to high CVH individuals. In addition, a significant decline in cognitive function Z-score was observed in individuals belonging to the low and moderate categories of CVH. Additionally, the proportion of moderate to severe depressive symptoms was discovered to be higher in these groups compared to individuals belonging to the high CVH.

**TABLE 1:** Baseline characteristics of the study population by cardiovascular health level (Weighted)*.

	Overall	Low CVH^†^	Moderate CVH^†^	High CVH^†^	*P*-value
Weighted *n*	43666424	2597171	28383489	12685765	
Age, *y*	69.06 (6.60)	66.42 (5.55)	69.19 (6.61)	69.32 (6.68)	< 0.001
Age level					0.047
60–69	1,235 (54.6)	121 (71.2)	836 (54.3)	278 (50.4)	
70–79	677 (29.9)	41 (24.1)	470 (30.5)	166 (30.1)	
80+	351 (15.5)	8 (4.7)	235 (15.2)	108 (19.6)	
Gender					0.610
Male	1,108 (49.0)	76 (44.7)	764 (49.6)	268 (48.6)	
Female	1,155 (51.0)	94 (55.3)	777 (50.4)	284 (51.4)	
Race					< 0.001
Mexican American	197 (8.7)	10 (5.9)	158 (10.3)	29 (5.3)	
Hispanic	217 (9.6)	18 (10.6)	151 (9.8)	48 (8.7)	
NH White	1,153 (51.0)	65 (38.2)	764 (49.6)	324 (58.7)	
NH Black	500 (22.1)	69 (40.6)	356 (23.1)	75 (13.6)	
Other Race	196 (8.7)	8 (4.7)	112 (7.3)	76 (13.8)	
Poverty ratio					< 0.001
< 1.3	538 (23.8)	71 (41.8)	381 (24.7)	86 (15.6)	
1.3–3.5	1,637 (72.3)	93 (54.7)	1,099 (71.3)	445 (80.6)	
> 3.5	88 (3.9)	6 (3.5)	61 (4.0)	21 (3.8)	
Education					< 0.001
Less than 9th grade	235 (10.4)	24 (14.1)	185 (12.0)	26 (4.7)	
9–11th grade	298 (13.2)	37 (21.8)	214 (13.9)	47 (8.5)	
High school graduate/GED or equivalent	531 (23.5)	49 (28.8)	386 (25.0)	96 (17.4)	
Some college/AA degree	652 (28.8)	49 (28.8)	434 (28.2)	169 (30.6)	
College graduate/above	545 (24.1)	11 (6.5)	320 (20.8)	214 (38.8)	
Don’t know	2 (0.1)	0 (0.0)	2 (0.1)	0 (0.0)	
Marital status					< 0.001
Coupled	1,295 (58.4)	84 (49.4)	878 (57.0)	360 (65.2)	
Single	941 (41.6)	86 (50.6)	663 (43.0)	192 (34.8)	
Depressive state					< 0.001
None/wild	2,059 (91.0)	134 (78.8)	1,392 (90.3)	533 (96.6)	
Moderate/above	204 (9.0)	36 (21.2)	149 (9.7)	19 (3.4)	
LE8 score	71.32 (13.01)	44.24 (4.46)	67.09 (7.84)	86.83 (40.06)	< 0.001
Cognition function (Z score)	1.10 (3.02)	0.41 (2.84)	0.85 (2.95)	1.77 (3.11)	< 0.001

CVH, cardiovascular health; NH, non-Hispanic; AA, associate degree.

*. Data were presented as weighted percentages or means.

^†^. Low CVH was defined as a LE8 score (out of 100 possible points) of 0–49, moderate CVH of 50–79, and high CVH of 80–100.

### 3.2 Contribution analysis of individual components of LE8 to overall score

In our study, the contributions of the various components of LE8 were quantified through regression analysis (Supplementary Table 2). The result demonstrates that all components reached statistical significance (*p* < 0.001) for their contributions to the overall health score. Sleep (β = 1.1729, 95% CI: 1.074–1.272) and diet (β = 1.1211, 95% CI: 1.019–1.223) emerged as the two most significant contributors among health behaviors. Nicotine Exposure (β = 1.0810, 95% CI: 0.989–1.173) and PA (β = 0.5337, 95% CI: 0.485–0.583) also showed significant contributions. As for health factors: BMI (β = 1.2084, 95% CI: 1.108–1.309) ranked as the most influential health factor, followed by blood glucose (β = 0.8845, 95% CI: 0.761–1.009) and lipid levels (β = 0.8773, 95% CI: 0.791–0.964). Blood pressure (β = 0.4093, 95% CI: 0.347–0.471), while statistically significant, had a relatively smaller contribution to the health score.

### 3.3 Associations of CVH with survival probability

**FIGURE 1:**
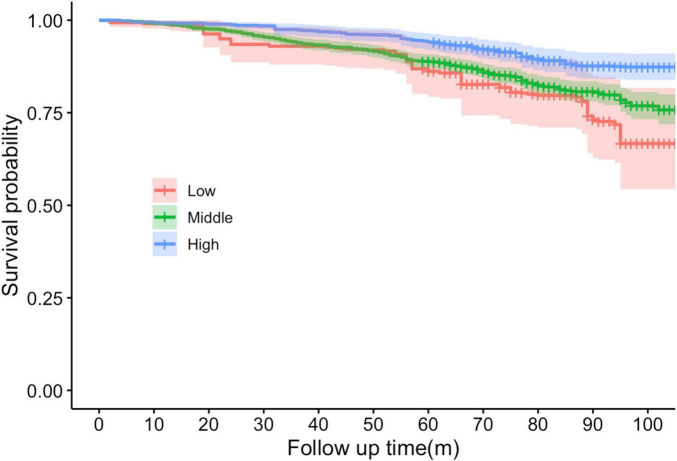
Kaplan–Meier survival curves stratified by cardiovascular health levels (Low, Middle, High) over the follow-up period. The shaded areas around each curve represent the 95% CIs. *X*-axis in the figure represents the follow-up time, which was measured in months in this study. *Y*-axis represents the probability of survival, ranging from 0 to 1.

The survival curves graph illustrates the probability of the population surviving over a specified period of follow-up (Figure 1). The *X*-axis in the figure represents the follow-up time, which was measured in months in this study. The *Y*-axis represents the probability of survival, ranging from 0 to 1, which depicts the proportion of individuals who did not die within a specific follow-up time in this study.

The red curve represents the group with a low CVH, and these individuals exhibit the most adverse survival prospects. The curves demonstrated the greatest decline, indicating the lowest probability of survival over time. The precipitous decline of the red line indicates that issues emerge at an early stage and recur frequently. The green curve represents a moderate CVH, and it can be observed that the probability of survival begins to decline after a period of time. This is a more gradual decline compared to the red line. The blue curve represents the high CVH group, whose curves remained the highest, flattest, and smoothest. This indicates that they persisted the longest throughout the observation period and had a higher chance of survival.

### 3.4 Associations of CVH with cognitive function

We calculated the variance inflation factor (VIF) for covariables in models separately. The VIF of all variables was less than 2, indicating no excessive multicollinearity problem (Wei et al., 2022). As shown in Table 2, for every ten points increment in LE8 score, the cognitive function of the regression model without adding any covariates improved by 0.42 (95% CI, 0.28–0.56) points, and in the adjusted model 1 and model 2, the cognitive function improved by about 0.23 (95% CI, 0.12–0.33) points and 0.21 (95% CI, 0.11–0.31) points. Further analysis of the discrepancies between different CVH groups revealed that when low CVH level was set as the reference group, cognitive function scores increased by 0.80 (95% CI, 0.12–1.47, model-1) and 0.70 (95% CI, 0.07–1.34, model-2) points in high CVH, respectively. These results were statistically significant, with all *P*-values less than 0.05 (Table 2).

**TABLE 2:** Association of the Life’s Essential 8 scores with cognition function Z score (Weighted).

	Univariable model		Multivariable model-1*		Multivariable model-2^†^	
	**β (95% CI)**	***P*-value**	**β (95% CI)**	***P*-value**	**β (95% CI)**	***P*-value**
**CVH levels**						
Low (0–49)	1 (Reference)	/	1 (Reference)	/	1 (Reference)	/
Moderate (50–79)	0.44 (−0.28 to 1.17)	0.223	0.35 (−0.26 to 0.96)	0.24	0.28 (−0.30 to 0.86)	0.42
High (80–100)	1.36 (0.50–2.23)	< 0.01	0.80 (0.12–1.47)	< 0.05	0.70 (0.07–1.34)	< 0.05
**LE8 scores (Continuous)**						
Per 10 points increase	0.42 (0.28–0.56)	< 0.001	0.23 (0.12–0.33)	< 0.001	0.21 (0.11–0.31)	< 0.001

CI, confidence interval; LE8, Life’s Essential 8.

*Adjusted for age (as a continuous variable), gender, race, poverty ratio, education levels, and marital status.

^†^Adjusted for age (as a continuous variable), gender, race, poverty ratio, education levels, marital status and depressive state.

Furthermore, a sensitivity analysis was conducted to excluding from the analysis any participants whose PHQ-9 scores indicated depression. The relationship between CVH and cognitive function persisted and was significant after removing subjects with PHQ-9 values over the depression threshold, although the effect size was slightly weakened (β = 0.338, *p* < 0.001).

### 3.5 Subgroup analyses of factors impacting the correlation of CVH with cognitive function

Table 3 shows the relationship between the CVH (continues LE8 score or levels) and cognitive function through subgroup analyses, which are stratified by age, gender, race and ethnicity, poverty ratio, marital status, and depressive state. *P*-value emphasizes whether the relationship between the independent variable and the dependent variable is significant in a particular subgroup. *P* for interaction focuses on whether this relationship is significantly different across subgroups.

**TABLE 3:** Associations of Life’s Essential 8 score for cognition function Z score grouped by gender, age, race and ethnicity, poverty ratio, marital status.

Subgroups	β (95% CI)	Cardiovascular health group			*P*-value	*P*-for interaction
		**Low CVH**	**Moderate CVH**	**High CVH**		
Gender						0.4
Male	0.16 (0.05–0.28)	Reference	0.61 (0.03–3.29)	0.77 (0.14–1.41)	0.006	
Female	0.2 (0.08–0.31)	Reference	0.26 (−0.28 to 0.8)	0.6 (0–1.21)	0.001	
Age						0.5
60–69	0.17 (0.07–0.28)	Reference	0.23 (−0.23 to 0.69)	0.53 (0–1.06)	0.001	
70–79	0.19 (0.03–0.34)	Reference	0.65 (−0.15 to 1.45)	0.79 (−0.07 to 1.65)	0.017	
80+	0.14 (−0.12 to 0.39)	Reference	−0.12 (−2.14 to 1.89)	0.31 (−1.74 to 2.36)	0.293	
Poverty ratio						0.03*
< 1.3	0.09 (−0.07 to 0.26)	Reference	−0.12 (−0.76 to 0.52)	0.25 (−0.57 to 1.08)	0.263	
1.3–3.5	0.19 (0.09–0.29)	Reference	0.64 (0.13–1.16)	0.86 (0.3–1.41)	0.000	
> 3.5	0.19 (−0.34 to 0.73)	Reference	1.6 (−1.04 to 4.25)	1.24 (−1.78 to 4.27)	0.48	
Marital status						0.16
Coupled	0.20 (0.1–0.31)	Reference	0.62 (0.07–1.17)	0.94 (0.34–1.53)	0.000	
Single	0.14 (0.01–0.26)	Reference	0.24 (−0.33 to 0.81)	0.34 (−0.32 to 1)	0.035	
Race and ethnicity						0.35
Mexican American	0.3 (0.02–0.58)	Reference	1.01 (−0.54 to 2.56)	1.37 (−0.34 to 3.08)	0.034	
Hispanic	0.17 (−0.08 to 0.42)	Reference	−0.08 (−1.22 to 1.05)	0.5 (−0.79 to 1.79)	0.195	
NH White	0.22 (0.1 to 0.34)	Reference	0.71 (0.05 to 1.37)	1.07 (0.36 to 1.78)	0.000	
NH Black	0.08 (−0.08 to 0.24)	Reference	0.36 (−0.25 to 0.97)	0.07 (−0.71 to 0.86)	0.332	
Other Race	0.02 (−0.24 to 0.29)	Reference	−0.32 (−1.96 to 1.32)	−0.3 (−2 to 1.39)	0.881	
Depressive state						0.55
None/wild	0.15 (0.06–0.23)	Reference	0.31 (−0.13 to 0.74)	0.51 (0.03–0.98)	0.001	
Moderate/above	0.29 (0.01–0.58)	Reference	0.54 (−0.42 to 1.51)	1.36 (−0.1 to 2.83)	0.045	

Each stratification was adjusted for age (continuous), gender, race and ethnicity, marital status, poverty income ratio, and educational level, except the stratification factor itself.

*P-value is less than 0.05, which is statistically significant.

We investigated the connection between CVH and cognitive function across various subgroups. These subgroups include factors such as β gender, age, poverty rates, marital status, race and ethnicity, and depression status. Results indicate that the relationship between CVH and cognitive function differs among these subgroups. Specifically, we found stronger associations in female (β = 0.2, *p*-value = 0.001) and individuals aged 70 to 79 (β = 0.19, *p*-value = 0.017). This was evident through higher β coefficient and significant *P*-value. However, no significant difference between groups was achieved compared with men and other age groups (*p* for interaction > 0.05). The results also showed that CVH on cognitive function remained consistent across all predefined subgroups except income level, with *P*-values greater than 0.05 for interactions. Whereas, when the poverty income ratio was employed, the *P* for interaction was less than 0.05 (*p* for interaction = 0.03), suggested that the positive effects of high CVH on cognitive function are different in different poverty levels.

### 3.6 Dose-response analysis of LE8 score with cognitive function

The *X*-axis represents the overall LE8 score for CVH, with larger scores representing higher levels of cardiovascular fitness. The *Y*-axis represents the β coefficient, which measures the association between CVH and cognitive function. The β coefficient shows the magnitude of change in cognitive function for each unit change in the CVH score. The 95% CI is displayed as a shaded area around the β coefficient line. This indicates the range of values within which the true beta coefficient is likely to fall, with 95% confidence.

**FIGURE 2:**
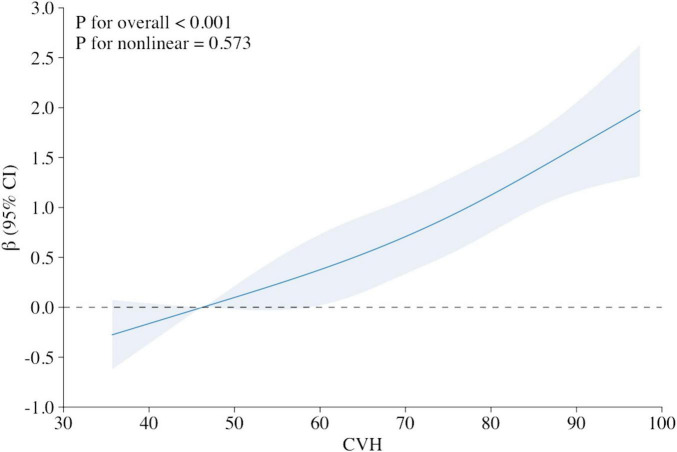
Relationship between cognitive function Z score and the Life’s Essential 8 score (stand for CVH), using restricted cubic spline regression model. Multivariable adjusted β (blue solid line) for the Life’s Essential 8 score’s relationship to cognitive performance, along with a 95% confidence interval (blue shaded region), adjusted for age, gender, race and ethnicity, poverty ratio, education and marital status. *X*-axis represents the overall LE8 score for CVH, with larger scores representing higher levels of cardiovascular fitness. *Y*-axis represents the β coefficient, which measures the association between CVH and cognitive function.

The multivariable adjusted RCS analyses revealed a linear association between LE8 score and cognitive function Z-score (*P* for overall < 0.001, *P* for nonlinear = 0.573; Figure 2).

### 3.7 Associations between CVH, depressive state, and cognitive function and the mediating effects of depressive state

As shown in Table 4, Pearson correlation analysis revealed correlation among CVH, depressive state and cognitive function. CVH was shown to be favorably correlated with cognitive function (*r* = 0.159, *p* < 0.001) and negatively correlated with depressed state (*r* = −0.234, *p* < 0.001). Cognitive function and depression were adversely correlated (*r* = −0.137, *p* < 0.001) (Table 4).

**TABLE 4:** Bivariate correlation matrix for cardiovascular health, depressive state, and cognitive function.

Variable	Cardio- vascular health	Depressive state	Cognitive function
Cardiovascular health	1		
Depressive state	−0.234***	1	
Cognitive function	0.159***	−0.137***	1

***p < 0.001.

As Table 5 illustrated, the ADE of CVH on cognitive function is substantial, quantified at 0.365 (95% CI, 0.33–0.40, *p* < 0.001). The Total Effect of CVH on cognitive function, combining both direct and mediated paths is 0.416 (95% CI, 0.375–0.45, *p* < 0.001). Depression as a mediating variable, the ACME representing the mediating effect was 0.051 (95% CI, 0.04–0.06) and the proportion mediated was 12.2% (*p* < 0.001) (Table 5).

**TABLE 5:** Intermediary effect analysis model.

	Observed coefficient	95% CI	*P*-value
Average direct effect	0.365	(0.33–0.40)	< 0.001
Average causal mediation effect	0.051	(0.04–0.06)	< 0.001
Total effect	0.416	(0.38–0.45)	< 0.001
Proportion mediated	0.122	(0.10–0.15)	< 0.001

## 4 Discussion

In this nationally typical cross-sectional investigation, we discovered a significant positive dose-response association between CVH, as represented by the LE8 score, and cognitive function in the senior population, presented as a linear association and remained significant after adjusting multiple variables (Table 2). And the result of the RCS also strengthens the credibility of this result (Figure 1). Survival analysis found that people with a high level of CVH represented by a high LE8 score had better survival prospects and a higher probability of survival, which is consistent with previous findings (Figure 2; Yi et al., 2023). Subgroup analyses showed the positive correlativity between LE8 scores and cognitive function was more prominent among participants who were younger, female, coupled, Mexican American, and moderate-to-severe depressed. People with normal or mild depression had a higher LE8 score than those with moderate to severe depression, suggesting some association between CVH and depressive states (Table 3). Correlation analysis results showed depression was significantly negatively correlate with CVH and cognitive function (Table 4), which is congruous with previous outcomes (Shen and Zou, 2024; Xian et al., 2024). The analysis of mediating variables of depression revealed depression demonstrated a notable mediating role in the relationship between CVH and cognitive function but the direct effect of cardiovascular health remained the primary pathway (Table 5). The sensitivity analysis results after excluding depression showed that the exclusion of depression did not affect the significance of the direct effect of CVH on cognitive function. On the whole, our findings accentuate the importance of maintenance of higher CVH and avoiding depression as means of prevention of cognitive decline.

Researchers frequently concentrate on the role of enhancement in one domain in enhancing the overall function. However, the factors influencing cardiovascular health and cognitive function are diverse and complex. Consequently, we selected two comprehensive test scores with recognition to measure the functionality of them. As mentioned earlier, to assess CVH, we selected the LE8 score, an enhanced measurement tool developed by the AHA and its partners that assesses the full range of lifestyle and physical indicators in the participants. To evaluate cognitive function, we selected a comprehensive assessment tool that is broadly applicable across diverse populations. In order to evaluate verbal fluency and semantic memory, participants in the AFT must name as many animals as they can in a certain amount of time. This exam is a reliable indicator of language proficiency, covering executive function and word retrieval. Previous study showed that cerebral blood flow is impacted by CVH, especially in the frontal lobes of the brain, which are linked to verbal fluency (Murata et al., 2010). The DSST assesses executive function, attention span, and processing speed. The DSST is a useful instrument for evaluating cognitive decline associated with CVH since processing speed is one of the cognitive areas that is typically affected by the condition (Walker et al., 2024). The CERAD Word List Learning Test tests both short-term and delayed memory by having participants learn and then recall a list of words. Given the correlation between memory impairment and hippocampus shrinkage and CVH, this test offers important new information on the connection between cardiovascular risk factors and memory decline (Shih et al., 2018, Kulshreshtha et al., 2019). Using Z-score allows us to combine the scores from the AFT, DSST, and CERAD word list learning tests into a comprehensive measure of cognitive function. This approach enables a more thorough assessment of how cardiovascular health impacts multiple cognitive domains.

Previous mechanism studies support the necessity and feasibility of analyzing the association between CVH and cognitive function. For example, Andrew R. Marks and his colleagues have found that heart failure causes the leakage of calcium ions in the endoplasmic reticulum (ER) in neurons in the brain, which leads to disruption of neurotransmission pathways and activation of various enzymes, leading to cognitive decline (Dridi et al., 2023). The CVH level in this study is defined by healthy lifestyle and health factors that have a positive effect on cognitive function and there are several relevant mechanism studies that support this relationship (Lloyd-Jones et al., 2022). Cognitive problems have a complicated and poorly understood etiology, with genetics, beta amyloid theory, tau protein theory and neuroinflammatory theory currently recognized (Torres et al., 2022, Zhang et al., 2024). It is worthwhile to investigate the complex and varied elements influencing cognitive function, as shown by prior research on the connection between blood heavy metal and cognitive function (Song et al., 2023). The findings of our investigation align with other studies on the connection between a healthy lifestyle and cognitive function in elderly individuals (Xian et al., 2024).

We found the level of CVH significantly affected the level of cognitive function (Table 2), which indicates that the indicators contained in LE8 have a certain effect on cognitive function. In the context of CVD, endothelial dysfunction stemming from hypertension, atherosclerosis, obesity, or metabolic syndrome can induce endothelial damage and inflammation (Horton and Barrett, 2021). This may reduce cerebral perfusion and lead to a decline in cognitive function, thereby corroborating our findings. Studies have shown that receiving daily vitamin supplementation may potentially act as a preventive measure in reducing cognitive decline and neurodegeneration in older adults (Harrison et al., 2014; Baker et al., 2023). Additionally, the appetite and digestive function of the elderly population are decreasing with the increase of age, and various bad eating habits will also affect cognitive function (Andriollo-Sanchez et al., 2005; Phillips, 2012). Given that some elderly individuals exhibit a diminished inclination to purchase healthcare products or possess limited capacity to assess product quality, enhancing their daily dietary intake quality could yield more significant benefits. Several policy measures may be implemented, including subsidizing farmers who cultivate fruits and vegetables, strengthening food safety oversight, and categorizing the sugar content in certain processed foods. It has been found that during PA, chemical signaling molecules produced by muscle cells stimulate hippocampal neurons, astrocytes regulate neuronal activity to prevent overexcitation, and both work together to co-ordinate the balance of brain function, thereby improving cognitive function (Lee et al., 2023). The increasing engagement of elderly individuals in various physical activities is noteworthy. This trend results from heightened health awareness within the population and government initiatives for widespread promotion. Furthermore, the regular organization of group activities across different regions may enhance motivation among seniors. Various harms caused by nicotine exposure, including cognitive impairment, have been extensively studied, for example, smoking can give rise to elevated oxidative stress and increased inflammation (Ge et al., 2020). But interestingly, recent studies have shown nicotine may reduce mortality and improve brain function, so whether it is friend or foe needs further research (Wang et al., 2023; Yang et al., 2023). Sleep is an important cause affecting cognitive function, and studies have reported that shorter (< 6 h) or longer (> 9 h) sleep duration and sleep fragmentation are related to cognitive decline, which is consistent with the scoring criteria for the sleep component in LE8 (Aakre et al., 2023). In our study, the contribution of sleep scores to the overall LE8 score ranks second (Supplementary Table 2). This indicates that sleep status is crucial for CVH. This finding aligns with research on the mechanisms by which sleep disorders lead to metabolic disruptions and cognitive decline (He et al., 2024). Besides, good sleep quality and circadian rhythms are associated with clearance of amyloid beta protein in the brain, and prolonged sleep increases the risk of occasional cognitive decline in cognitively normal adults (Musiek et al., 2015; Suh et al., 2018). Previous research indicates that darkness plays a vital role in melatonin synthesis, essential for sleep (Claustrat and Leston, 2015). Urbanization, particularly city lights at night and ubiquitous mobile phone usage, can adversely affect melatonin secretion. Consequently, the younger generation should consider enhancing the sleep environment for the elderly.

As for the mechanism between the health factors part of LE8 and cognitive function. Among them, the BMI score had the greatest influence on the overall LE8 score (Supplementary Table 2). Weight gain was discovered to be associated with a decrease in episodic memory, and waist-to-hip ratio was recommended over BMI (Hartanto et al., 2019). There are also studies showing that new indicators that can better predict the relationship between weight and CVD risk which may be updated in the future to measure obesity (Cai et al., 2023; Zhao et al., 2024). Prior research indicates that individuals with higher body mass often experience increased levels of blood pressure, lipids, and glucose (Cercato and Fonseca, 2019). Furthermore, in conjunction with our findings, we determine that weight loss within a normal range may provide more significant CVH advantages compared to pharmacological management of these three blood markers. Hyperglycemia raises the risk of cognitive decline and can cause harm to the nervous system. The mechanisms involved include synaptic dysfunction, neuronal apoptosis, oxidative stress and neuroinflammation. It is believed that metabolic disorders represented by impaired glucose metabolism and insulin resistance are significant (Gupta et al., 2023). Thus, it is essential to regulate sugar consumption in a time when sugary products are plentiful. Establishing guidelines for the permissible sugar levels or explicitly outlining sugar content standards can help decrease the chances of unintentional excessive sugar intake. Hyperlipidemia is more common in middle-aged and elderly people, who are at high risk for cognitive decline (Yang et al., 2020). For example, when low-density lipoprotein is high, it will accumulate the arterial walls of blood vessels in the heart and brain, gradually forming atherosclerotic plaque that obstructs the corresponding blood vessels. At the same time, it also increasing the inflammatory response, leading to white matter lesion and cognitive decline (Smit et al., 2016). Hypertension has been widely (Cai et al., 2024). For the past years, the relationship between hypertension and cognitive function has received considerable attention from epidemiological researchers, with recent evidence suggesting that a relation between cognitive impairment and hypertension, including its severity. Additionally, researchers have identified a link between elevated arterial stiffness as determined by pulse wave velocity and lower cognitive function. These studies suggest vascular stiffness could be a contributing factor to dementia and cognitive decline (Muela et al., 2018). However, a study found that middle-aged hypertension is widely associated with cognitive impairment but well-managed hypertension in older adults may not necessarily lead to a decline in cognitive abilities (Tadic and Cuspidi, 2022). This suggests that while hypertension can cause cognitive impairment, other health conditions and overall management of hypertension may alleviate or even counteract its effects on cognition. This indicates that research on hypertension related to cognitive impairment should broadly include more influencing factors. In short, a diet low in oil, fat, and salt proves more advantageous for the elderly demographic. Continued investigation into specific dietary patterns will be crucial in formulating tailored nutritional guidelines that promote longevity and overall health among older adults. These findings offer a solid theoretical foundation for this research. These results establish a robust theoretical framework for this research and offer insights for diverse perspectives in public health discourse.

Depression is a common psychiatric disorder that results from a complex interplay of social, psychological and biological factors (Monroe and Harkness, 2022). Although there is no clear evidence that cerebrovascular lesions can directly lead to depression, some studies believe that vascular brain injury is a crucial risk factor for depression in later life (Allan et al., 2012). Other mechanisms include dysregulation of the autonomic nervous system, dysfunction of hypothalamic-pituitary-adrenal axis and endothelial dysfunction (Schiepers et al., 2005; Janssen et al., 2021). Cerebrovascular lesions (white matter lesions, WML) due to risk factors such as hypertension compromise the integrity of frontal striatal circuits that regulate mood and executive function, which may be a mechanism (Vicario and Cerezo, 2023). Studies have shown that higher BMI are more likely to lead to depression, regardless of other health problems caused by obesity, and this phenomenon affects women more (Tyrrell et al., 2019). At the same time, the presence of depression also increases the risk of CVD (Li et al., 2019). Furthermore, we found that CVH containing lifestyle habits is directly associated with depressive status, which is consistent with prior findings that poor lifestyle habits are common in patients with affective disorders (Musselman et al., 1998). Some previous studies could explain the underlying mechanism. For example, physical activities promote DA secretion, and substantia nigra compacta (SNc) DA neurons project to the striatum, which in turn releases DA (Matsuda et al., 2009). These DAs play a significant role in exercise, motor learning, reward, motivation, and mood to maintain a positive state of mind (Threlfell et al., 2012). Excessive exposure to nicotine may lead to addiction, making people more sensitive and anxious (Kolla et al., 2023). In addition, a good diet and a variety of nutrients can help improve the health of the human body, for example, tyrosine and tryptophan in protein have a positive effect on improving depression (Yun et al., 2021). The influence of sleep quality and rhythm on depression is obvious, and the related mechanisms include the decrease of circadian oscillation activity associated with aging, the decrease of melatonin secretion, and the functional changes of neural circuits (Dong et al., 2022; Yao et al., 2024).

Depression is often accompanied by a loss of interest in activities and refusal of social activities, which may lead to a decline in cognitive functioning (van der Linde et al., 2014; Kelly et al., 2017). The mechanisms linking depression and cognitive dysfunction are multifaceted, involving neurobiological, psychological, and environmental factors. In terms of neurotransmitters, alterations in serotonergic neuronal pathways have been associated with emotion regulation and cognitive processes, particularly memory and executive function, while dopaminergic deficits have been associated with reduced motivation, executive dysfunction and attention deficits (Schapira et al., 2017; Smith et al., 2023). Additionally, depression is associated with reduced hippocampal volume and neuroinflammation, affecting memory and learning, and amygdala hyperactivity is associated with negative emotion processing (Nolan et al., 2020; Li et al., 2023).

Several studies showed that inflammation serves as a critical link among cardiovascular disease, cognitive decline, and depression. Chronic inflammation tied to obesity and cardiovascular issues has shown to foster neuroinflammation (Swanson et al., 2012). This factor significantly influences the pathophysiology behind cognitive decline and depressive symptoms. Increased levels of pro-inflammatory cytokines, including interleukin-6 (IL-6) and tumor necrosis factor-alpha (TNF-α), appear in patients with cardiovascular ailments. Moreover, these cytokines contribute to hippocampal atrophy and neurodegeneration, which are foundational to cognitive impairment and depression (Miller and Raison, 2016). Additionally, metabolic syndrome, marked by insulin resistance, dyslipidemia, and obesity, poses a collective risk for cardiovascular disease, cognitive decline, and depression. Notably, insulin resistance correlates with diminished cerebral glucose metabolism. This decline negatively impacts cognitive function over time (Woodfield et al., 2022). Furthermore, insulin resistance interrupts neurotransmitter equilibrium, particularly within serotonin and dopamine pathways, thereby elevating the risk of depression (Sarwar et al., 2022). These studies demonstrate that the interplay between cardiovascular health, cognitive function, and depression is intricate and layered. Factors such as vascular dysfunction, inflammation, oxidative stress, metabolic disorders, neurotransmitter imbalances, and psychosocial issues all play a role in the onset and advancement of cognitive and emotional disorders linked to cardiovascular disease. In this study, analyzing depression’s mediating role may offer insights for future discussions regarding its intermediary position in the relationship between cardiovascular disease and cognitive decline. According to our research, there may be some partial mediation of the relationship between cognitive function and CVH by depressed state. In particular, a high level of CVH may prevent the emergence of depressive symptoms, thereby lowering the risk of cognitive decline. This is supported by the results that the direct effect of CVH on cognitive function was 0.365, the total effect was 0.416, and the indirect effect mediated by depressive symptoms was 0.051 (Table 5). These results suggest that paying attention to the cardiovascular health of the elderly might enhance their mental and cognitive functioning in addition to lowering the incidence and mortality of cardiovascular illnesses. Future cohort studies may evaluate the effect of early intervention with cardiovascular health on emotional health and cognitive function in aging populations. By employing longitudinal designs, researchers can capture changes over time, providing a deeper understanding of the interplay between cardiovascular health and psychosocial factors in senior citizens. Improving the CVH of the elderly population in accordance with the recommended measures in the corresponding guidelines or government documents can have multiple benefits, thereby improving the quality of life. Therefore, all social security departments should actively promote public health and medical measures to maintain and enhance the CVH of the population. At the same time, subgroup analysis results indicate that the beneficial impacts of high CVH levels vary significantly among individuals with different income levels (Table 3), which suggests the need to account for socioeconomic status when developing interventions.

The LE8 index employed in this study is a comprehensive index designed to evaluate the relationship between CVH and cognitive function from a multitude of perspectives, underscoring the necessity to consider not only physical indicators but also lifestyle habits as equally significant. In addition, the NHANES data selected in this study were collected using a complex, multi-stage probability sampling design with non-institutional resident population serving as the representative sample. This approach ensured the acquisition of high-quality data. A weighted analysis and controlled for many confounding factors was also used in the data analysis, so the results obtained in this study are reliable when applied to the US non-institutional civilian. And there are some limitations. First of all, the lack of some data inevitably leads to the limitation of the survey sample size. Secondly, the data of life factors were obtained through questionnaire survey, which may have some limitations of authenticity. And antidepressant use was not taken into account in the evaluation of depression due to limited data. Finally, the specificity and sensitivity of LE8 index to cardiovascular health level still need more investigation methods to determine.

## 5 Conclusion

Findings from this nationally representative sample of Americans demonstrate a significant positive relationship between CVH and cognitive function. Specifically, an increase in the LE8 score, which represents cardiovascular health, was associated with an increased likelihood of cognitive function, and depression played a negative role in this. These conclusions suggest that the promotion of healthy lifestyle, improvement of cardiovascular indicators, and mental health counseling can be considered in the development of measures and treatment to improve cognitive function in older adults. Through formulating appropriate diet and exercise programs for the elderly, encouraging the supervision of smoking cessation, regular physical examination, supervision of taking medicine on time and other measures to achieve the goal of healthy aging.

## Data Availability

The datasets presented in this study can be found in online repositories. The names of the repository/repositories and accession number(s) can be found below: https://www.cdc.gov/nchs/nhanes/.
