# Supplementary material for: Association of cardiovascular health with cognitive function and the mediating effect of depressive state
Source: Front Aging Neurosci. 2024 Dec 18;16:1465310. doi: 10.3389/fnagi.2024.1465310 (PMC11688292; doi:10.3389/fnagi.2024.1465310)
Supplement: Supplementary file 1 [file Table_1.docx]

Supplementary Table1 Life’s Essential 8 components and grading rules.

| Life’s essential 8 components | Method of measurement | Quantification of CVH metric | Score, points |
| --- | --- | --- | --- |
| Diet | Quantiles of HEI-2015 (population) | 1st–24th  25th–49th  50th–74th  5th–94th  ≥95th | 0  25  50  80  100 |
| PA | Self-reported minutes of moderate or vigorous PA per week | 0  1–29  30–59  60–89  90–119  120–149  ≥150 | 0  20  40  60  80  90  100 |
| Nicotine exposure | Self-reported use of cigarettes or inhaled NDS | Current smoker Former smoker quit <1 y, or currently using inhaled NDS, with active indoor smoker  Former smoker quit <1 y, or currently using inhaled NDS, without active indoor smoker  Former smoker, quit 1–<5 y, with active indoor smoker  Former smoker, quit 1–<5 y, without active indoor smoker  Former smoker, quit ≥5 y, with active indoor smoker  Former smoker, quit ≥5 y, without active indoor smoker  Never smoker, with active indoor smoker  Never smoker, without active indoor smoker | 0  5  25  30  50  55  75  80  100 |
| Sleep | Self-reported average hours of sleep per night | <4  4–<5  5–<6 or ≥10  6–<7  7–<9  9–<10 | 0  20  40  70  90  100 |
| BMI | Body weight (kilograms) divided by height squared | ≥40.0  35.0–39.9  30.0–34.9  25.0–29.9  <25 | 0  15  30  70  100 |
| Blood lipids | Plasma total and HDL cholesterol with calculation of non–HDL cholesterol | ≥220 or 190–219 (take medication)  190–219 (no medication) or  160–189 (take medication)  160–189 (no medication) or  130–159 (take medication)  130–159 (no medication)  <130 (take medication)  <130 (no medication) | 0  20  40  60  80  100 |
| Blood glucose | Casual HbA1c (%) | Diabetes with HbA1c ≥10.0  Diabetes with HbA1c 9.0–9.9  Diabetes with HbA1c 8.0–8.9  Diabetes with HbA1c 7.0–7.9  Diabetes with HbA1c <7.0  No diabetes and HbA1c 5.7–6.4  No history of diabetes and HbA1c <5.7 | 0  10  20  30  40  60  100 |
| BP | Appropriately measured systolic and diastolic BPs | ≥160 or ≥100  140–159 or 90–99 (take medication)  140–159 or 90–99 (no medication)  130–139 or 80–89 (take medication)  130–139 or 80–89 (no medication)  120–129/<80 (take medication)  120–129/<80 (no medication) <120/<80 (take medication)  <120/<80 (no medication) | 0  5  10  30  50  55  75  80  100 |

CVH, cardiovascular health; HEI, Healthy Eating Index; PA, physical activity; NDS, nicotine-delivery system; BMI, body mass index; HbA1c, hemoglobin A1c; HDL, high-density lipoprotein; BP, blood pressure.

Supplementary Table2 Analysis of the contribution of different components of Life’s Essential 8 to the overall score. Weighted.

| LE8 components | β(95%CI) | *P* value |
| --- | --- | --- |
| Diet | 1.1211 (1.019-1.223) | < 0.001 |
| PA | 0.5337 (0.485-0.583) | < 0.001 |
| Nicotine exposure | 1.0810 (0.989-1.173) | < 0.001 |
| Sleep | 1.1729 (1.074-1.272) | < 0.001 |
| BMI | 1.2084 (1.108-1.309) | < 0.001 |
| Blood lipids | 0.8773 (0.791-0.964) | < 0.001 |
| Blood glucose | 0.8845 (0.761-1.009) | < 0.001 |
| BP | 0.4093 (0.347-0.471) | < 0.001 |

PA, physical activity; BMI, body mass index; BP, blood pressure.

Note: β represents the estimated regression coefficient for each component.
